# Oncolytic viruses in head and neck cancers: clinical applications and therapeutic potential

**DOI:** 10.3389/fmicb.2025.1641267

**Published:** 2025-08-13

**Authors:** Aditi Gupta, Samiran Ranjit Chavan, Ravisekhar Gadepalli, Puneet Pareek

**Affiliations:** ^1^Department of Microbiology, All India Institute of Medical Sciences, Jodhpur, India; ^2^Department of Radiation Oncology, All India Institute of Medical Sciences, Jodhpur, India

**Keywords:** oncolytic viruses, head and neck squamous cell carcinoma, talimogene laherparepvec, immunotherapy, cancer virotherapy, combination therapy

## Abstract

**Background:**

Head and neck squamous cell carcinoma remains a significant global health burden, particularly in low-resource regions like India. Conventional treatments often fall short in achieving durable responses, prompting the need for novel therapies.

**Objective:**

This review outlines the clinical progress, mechanism of action, and emerging therapeutic potential of oncolytic viruses (OVs) in the management of HNSCC, with an emphasis on ongoing trials, approved agents, and future directions.

**Methods:**

Data were extracted from published literature on PubMed and ClinicalTrials.gov. Focus was placed on mechanisms of viral action, regulatory approvals, trial outcomes, and rational combinations with existing therapies.

**Findings:**

Oncolytic viruses exert dual antitumor effects through selective viral replication and immune system activation. Talimogene laherparepvec (T-VEC) remains the only FDA-approved OV, while others like Oncorine and Teserpaturev show regional efficacy. Multiple early-phase trials are underway evaluating OV combinations with checkpoint inhibitors, chemotherapy, and radiotherapy. Although clinical responses have been encouraging, challenges such as tumor penetration, immune clearance, and hypoxic environments remain.

**Conclusion:**

Oncolytic virotherapy holds considerable promise in HNSCC. Advances in virus design, delivery platforms, and personalized approaches are essential for transitioning this modality from experimental settings into routine clinical practice.

## Introduction

Head and neck cancer is the sixth most common cause of cancer worldwide accounting for approximately 5% of all malignancies (Bray et al., 2024). In India, head and neck cancer is a major public health problem comprising 26% of all cancers in males and 8% in females (Bagal et al., 2023). Squamous cell carcinoma accounts for around 90% of the cases with the most common sites being the oral cavity, pharynx, larynx, nasopharynx and paranasal sinuses. The current treatment options mainly include surgery, radiotherapy and chemotherapy in differing sequences and combinations. Surgery is the standard of care for most oral cavity cancers with radiotherapy playing an integral role in the adjuvant setting. For inoperable cases or sites, combination chemoradiotherapy has emerged as a promising curative option while in cases of recurrent or metastatic disease, salvage surgery, re-irradiation and chemotherapy can be used in varying combinations to optimize disease control and survival. In recent years, targeted therapy and immunotherapy have gained prominence, especially with immune checkpoint inhibitors aiming to restore tumor-directed immune activity. However, these newer options remain expensive and inaccessible to many patients in low-resource countries, which highlights the need for cost-effective immunotherapy alternatives. Despite recent advances in treatment, head and neck cancer confers significant morbidity for the patient and society with 40%–60% patients ultimately suffering from local recurrence or distant metastases. The role of the local and systemic immune environment in disease progression and treatment resistant tumors has drawn attention to novel biological therapies, including oncolytic viruses. These uniquely combine direct tumor lysis with immune system modulation. Their biological origin and virological mechanisms offer a distinct advantage in selectively targeting cancer cells. In high burden resource constrained settings, microbiological driven treatment strategies offer a promising avenue.

## Methodology

This mini-review was conducted by searching the PubMed and ClinicalTrials.gov databases using the terms “oncolytic virus” AND “head and neck cancer.” Only articles published in English were included. No restrictions were applied to publication date or study type. Additional references were identified through citation tracking of relevant reviews and primary studies. The most recent and clinically significant trials, regulatory updates, and insights were prioritized for inclusion.

## Mechanism of action of oncolytic viruses

Cancer therapy has evolved significantly, transitioning from surgery and radiotherapy to the incorporation of chemotherapy and, more recently, immunotherapy. While immunotherapy has seen a surge in clinical interest, historical reports of spontaneous tumor regression during viral infections trace the origins of this concept back to the early 19th century (Dobosz and Dzieciątkowski, 2019). The usage of microorganisms and vaccines to treat cancer is not entirely unknown, with Bacillus Calmete Guerin (BCG) vaccine being successfully used to treat bladder cancer (Oiseth and Aziz, 2017).

Oncolytic viruses refer to those viruses which either occur naturally or are genetically engineered to enhance antitumor activity by actively replicating inside the host (Sanmamed and Chen, 2018). The first oncolytic virus to be approved by Food and Drug Administration (FDA) was for malignant melanoma named as talimogene laherparepvec (T-VEC), a genetically modified herpes simplex virus (Zhang et al., 2023). Since then, the promise of oncolytic viruses as a means of immunotherapy has captured the interest of many. Many ongoing trials are in progress for the development and successful administration of oncolytic virotherapy in various cancers. In head and neck cancers, trials have been conducted with genetically modified adenovirus, coxsackie virus, vaccinia and measles (Spirito et al., 2024).

Oncolytic viruses act through two principal mechanisms: direct oncolysis and immune activation. Direct tumor lysis occurs through selective replication in cancer cells, which is facilitated by the altered molecular landscape of tumors–such as upregulated viral receptors, metabolic reprogramming, and impaired interferon signaling pathways (Kaufman et al., 2015). These mechanisms represent an exploitable weakness in the tumor’s virological defense, offering a microbiologically precise avenue for therapeutic intervention. This tumor-selective tropism is further enhanced by mutations in tumor suppressor genes and disrupted antiviral defense mechanisms (Rahman and McFadden, 2021).

**FIGURE 1:**
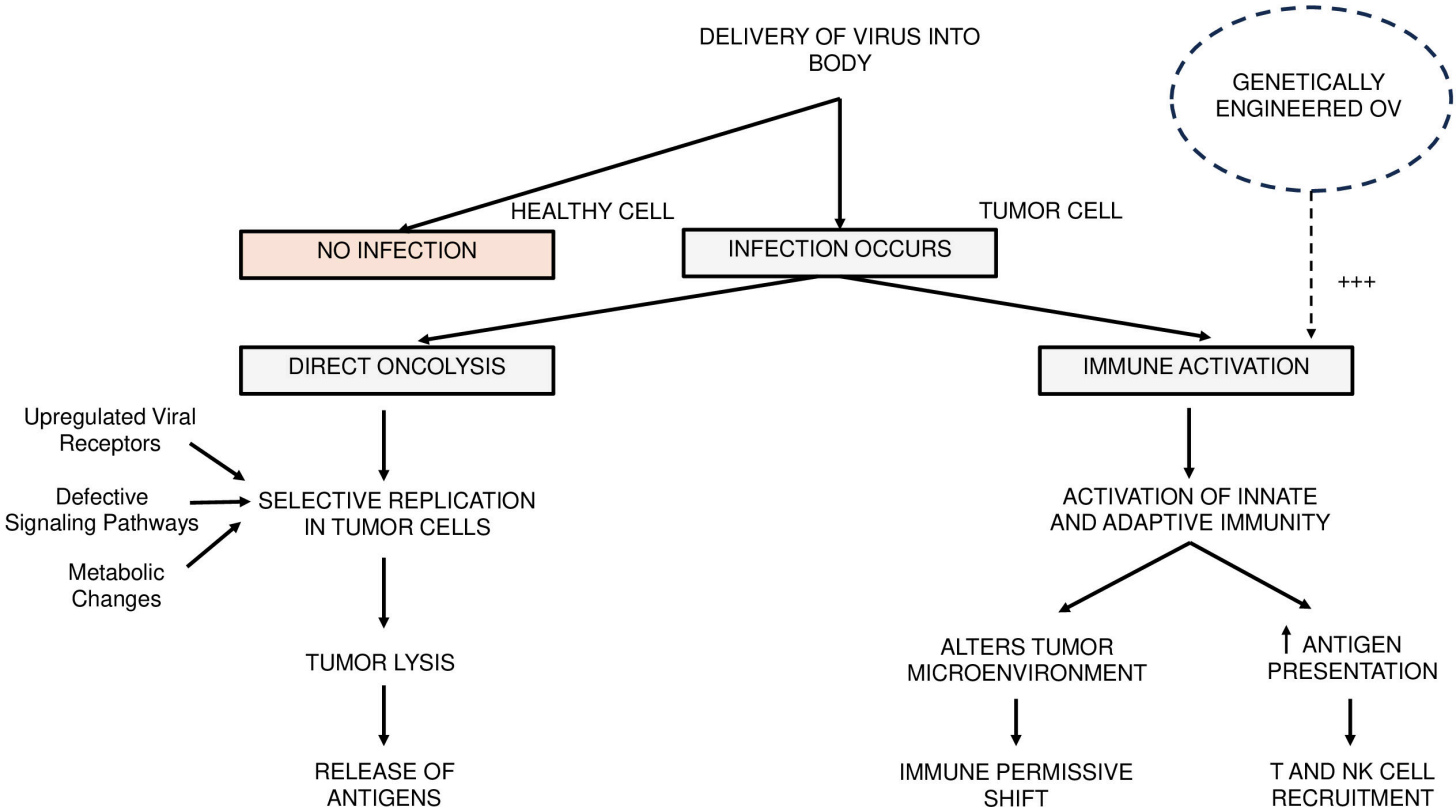
Mechanism of action of oncolytic viruses in cancer therapy. This schematic illustrates the dual mechanisms through which oncolytic viruses (OVs) exert antitumor effects in head and neck squamous cell carcinoma. Following the selective infection of tumor cells, OVs replicate preferentially due to tumor-specific vulnerabilities, such as upregulated viral receptors, defective signaling pathways, and metabolic alterations. This leads to tumor cell lysis and release of antigens. Antigen release contributes to the activation of innate and adaptive immune responses, promoting antigen presentation and recruitment of T and NK cells. The tumor microenvironment is concurrently altered to a more immunopermissive state. Genetically engineered OV’s may further enhance these effects, indicated by a +++ symbol in the figure, through the expression of immunostimulatory transgenes. +++ indicates enhanced immune activation through genetic engineering.

Upon infection, OVs hijack the cellular protein synthesis machinery to produce viral progeny, ultimately leading to cell death. Tumors often evade immune detection by suppressing local immune responses and altering immune cell function. However, OVs can counteract these effects by activating both innate and adaptive immunity, enhancing antigen presentation, and transforming the immunosuppressive tumor microenvironment into one that is immunologically active. The dual induction of antiviral and antitumor immunity represents a unique combination of virology and immunotherapy – distinct from traditional cytotoxic or targeted agents. A schematic overview of these mechanisms is illustrated in Figure 1 highlighting the pathways through which OV’s have anti-tumor activity.

Genetic engineering of OVs enables insertion of immunostimulatory genes like cytokines (or tumor-specific promoters, improving both the therapeutic index and tumor-targeting specificity. Despite these advancements, efficient delivery remains a significant hurdle, particularly due to challenges in localized delivery and immune-mediated clearance. Promising strategies to enhance OV delivery include the use of cellular carriers, such as T cells, tumor cells, myeloid-derived suppressor cells, and mesenchymal stem cells (Peng et al., 2024; Bai et al., 2019).

## FDA-approved and investigational oncolytic viruses

Several OVs are currently undergoing clinical development, although only one–T-VEC –has been approved by the United States FDA to date. T-VEC is a genetically engineered herpes simplex virus type 1 (HSV-1), designed to express human granulocyte-macrophage colony-stimulating factor (GM-CSF) while carrying deletions in the ICP34.5 and ICP47 genes. These deletions attenuate viral virulence and enhance antigen presentation by promoting immune system activation (Zhang et al., 2023). Trials are also being conducted to explore its potential in the treatment of other cancers such as pancreatic cancer and basal cell carcinoma (Runcie et al., 2025; Ressler et al., 2025).

In addition to T-VEC, several OVs have been approved for clinical use in specific countries outside the United States, demonstrating varying regulatory approaches worldwide.

The first oncolytic virus to be approved for cancer treatment anywhere in the world was Oncorine (H101) in China. Approved in 2005 by the Chinese FDA, it is indicated for nasopharyngeal carcinoma in combination with chemotherapy (Liang, 2018). Oncorine is a genetically modified adenovirus with a deletion in the E1B 55kDa gene, which allows it to selectively replicate in p53-deficient tumor cells. In normal cells, E1B-55kDa binds and inactivates p53 to allow viral replication. By deleting this gene, Oncorine can only replicate in cells where p53 is already non-functional, a common feature observed in cancer cells. This ensures that it multiplies only in tumor tissue and spares the normal cells. While initially approved for nasopharyngeal cancer, reports of its clinical benefit have also been published for other tumor types (Zhang J. et al., 2021; Zhang J. et al., 2021).

Rigvir, another early oncolytic virus, was approved in Latvia in 2004, and is also registered in Georgia, Armenia, and Uzbekistan. It is derived from the Wallace strain of ECHO-7, a non-genetically modified enterovirus of the Picornaviridae family (Alberts et al., 2018). Adapted specifically for melanoma, Rigvir is believed to function via cytolytic activity. However, its mechanism of action remains incompletely understood, and its clinical efficacy remains controversial due to limited data from well-controlled clinical trials (Hietanen et al., 2022).

In Japan, the oncolytic virus Teserpaturev (G47Δ, brand name Delytact) was granted conditional approval in 2021 for the treatment of glioblastoma, making it the first OV approved for any central nervous system (CNS) malignancy. This genetically modified virus contains deletions in ICP34.5, ICP6, and ICP47 genes, which increase selectivity for tumors and improve overall safety (Ghorab et al., 2025). Table 1 summarizes currently approved oncolytic viruses, their viral platform, regions of approval, indications, and key genetic modifications.

**TABLE 1:** Currently approved oncolytic viruses across the globe.

Virus name (brand)	Virus type	Country/region of approval	Year	Indication	Genetic modification	Notes
Talimogene laherparepvec (T-VEC)	HSV-1	United States, EU	2015	Unresectable melanoma	Deletions in ICP34.5 and ICP47; insertion of GM-CSF	First FDA- and EMA-approved oncolytic virus; intratumoral injection
Oncorine (H101)	Adenovirus (Ad5)	China	2005	Nasopharyngeal carcinoma + chemotherapy	E1B 55kDa gene deleted	First OV ever approved; replicates in p53-deficient tumors
Rigvir	ECHO-7 enterovirus	Latvia, Georgia, Armenia, Uzbekistan	2004	Melanoma	None (non-GMO; adapted via selection)	Mechanism unclear; clinical data limited and efficacy debated
Teserpaturev (Delytact)	HSV-1 (G47Δ)	Japan	2021	Glioblastoma	ICP6, ICP34.5, ICP47 deleted	Conditionally approved; first OV approved for CNS malignancy

While several oncolytic viruses have received regulatory approval in specific countries, these approvals apply to malignancies outside the scope of classical head and neck squamous cell carcinoma. For example, Oncorine is approved in China exclusively for nasopharyngeal carcinoma which exists as a distinct clinical and biological entity and differs from conventional HNSCC (Liang, 2018; Cantù, 2023). Agents like T-VEC, Rigvir, and Teserpaturev have received approvals for melanoma or glioblastoma, but not for head and neck malignancies typically seen in India and other low- and middle-income countries. Oral cavity cancers constitute the most prevalent form of HNSCC in India, bringing to notice a significant mismatch between global regulatory developments and regional disease burden (Bagal et al., 2023; Goyal et al., 2025) This highlights the critical need for context-specific research and clinical trials to evaluate the role of oncolytic virotherapy in classical HNSCC populations, particularly within LMIC settings.

## Clinical trials in head and neck cancer

The clinical development of OVs in HNSCC remains in an early stage. Globally, numerous studies have been initiated to evaluate OV safety, dosing, and preliminary efficacy in this setting. These agents follow the standard clinical trial trajectory, progressing from early Phase I safety trials to Phase II efficacy assessments and, where applicable, into Phase III randomized studies. However, the majority of trials in HNSCC remain at the Phase I/II stage, with a notable paucity of large-scale Phase III or IV evaluations (Sedgwick, 2014).

The current clinical trial landscape for oncolytic virotherapy in head and neck malignancies reveals a predominance of early phase clinical trials, with limited Phase III and IV data available. A comprehensive search of the ClinicalTrials.gov database using the terms “head and neck cancer” and “oncolytic” identified multiple ongoing studies, which are summarized in Table 2. This distribution reflects the relatively nascent stage of oncolytic virus development in this specific cancer population, with many Phase I and II trials actively recruiting participants.

**TABLE 2:** Currently recruiting clinical trials of oncolytic viruses in head and neck squamous cell carcinoma.

Virus/platform	Trial ID	Phase	Therapy strategy	Trial status	Country/region	Sponsor
Adenovirus	NCT06549946	I/II	Ixovex + Pembrolizumab	Recruiting	London, United Kingdom	Psivac Ltd.
Vaccinia	NCT06444815	I	VET3-TGI – initially followed by pembrolizumab	Recruiting	Multiple locations in the United States	KaliVir Immunotherapeutics
Adenovirus	NCT06265025	I/II	GM103 – initially followed by pembrolizumab	Recruiting	Seoul, South Korea	GeneMedicine Co., Ltd.
Adenovirus	NCT05222932	I	TILT-123 in combination with avelumab	Recruiting	Finland	TILT Biotherapeutics Ltd.
Adenovirus	NCT05076760	I	MEM-228 combined with nivolumab	Recruiting	Florida and North Carolina, United States	Memgen, Inc.
Adenovirus	NCT03740256	I (first in-human)	CAdVEC in combination with HER2 specific CAR T cells	Recruiting	Texas, United States	Baylor College of Medicine
Vaccinia	NCT06910657	I (first in-human)	Safety of IDOV-immune	Active, not yet recruiting	United States and Australia	ViroMissile, Inc.
Herpes simplex virus	i) NCT05961111 ii) NCT05830240	I	Safety of R130	Recruiting	Shandong, China	Shanghai Yunying medical technology

Among the approximately ten completed or terminated studies investigating oncolytic virotherapy for head and neck cancers, only two have published results. The first trial evaluated OBP-31, a genetically engineered adenovirus administered in combination with stereotactic body radiation therapy (SBRT) and pembrolizumab (NCT04685499). This study enrolled a single participant before termination due to insufficient patient recruitment. The second completed trial examined PF-07263689, a vaccinia virus construct, in combination with sasanlimab (NCT05061537). This investigation was discontinued for business-related reasons rather than safety or efficacy concerns, as documented in the trial registry.

Notable contributions to the field include a Phase I study combining oncolytic GM-CSF with cisplatin, which demonstrated promising tumor response rates (Harrington et al., 2010). The same investigative team subsequently initiated the MASTERKEY-232 trial, evaluating T-VEC in combination with pembrolizumab through planned Phase Ib and Phase III components (Harrington et al., 2020). However, progression to the Phase III portion was ultimately not pursued after Phase Ib results showed comparable efficacy to pembrolizumab monotherapy, suggesting limited additive benefit from the combination approach. The modest efficacy observed in the MASTERKEY-232 trial may be partially attributed to the lack of biomarker-driven patient stratification. Including factors such as PD-L1 expression, HPV status, and baseline tumor-infiltrating lymphocyte (TIL) density in future studies could help better identify patients most likely to benefit from combination therapies. Moreover, the therapeutic impact of T-VEC may have been limited by suboptimal intratumoral delivery, as responses were mostly confined to injected or superficial lesions, with minimal effect at distant metastatic sites.

A gap in the global trial landscape is the absence of dedicated OV trials in India or similar LMIC contexts, despite these regions carrying a high share of the HNSCC burden. Without inclusion of diverse populations in trial design, findings may lack validity for non-Western settings. In addition, infrastructural, funding, and regulatory constraints contribute to this void, which in turn delays the availability of such therapies in resource-constrained settings.

## Combination of oncolytic viruses with other modalities

Monotherapy with OVs has demonstrated limited efficacy in solid tumors, including HNSCC, due to tumor heterogeneity, intrinsic immune resistance, and barriers within the tumor microenvironment (Zou et al., 2022).

Therefore, combining OVs with other established therapeutic modalities–such as chemotherapy, immune checkpoint inhibitors (ICIs), and radiotherapy–is being increasingly investigated as a strategy to enhance therapeutic outcomes. Combination therapies are advantageous because they often exhibit non-overlapping toxicities and reduced cross-resistance. In HNSCC, where most patients receive chemotherapy in the adjuvant or recurrent setting, integrating OVs into existing regimens offers a logical treatment extension.

One approach explores the combination of chemotherapeutic drugs with oncolytic viruses. In 2005, China approved Ad-H101, an oncolytic adenovirus for head and neck cancer after phase III studies showed a higher response rate for Ad-H101 plus chemotherapy with 5-FU (79%–72%) compared to chemotherapy alone (40%). Various drugs such as cyclophosphamide, cisplatin, gemcitabine, 5-FU, doxorubicin, taxanes, histone deacetylase inhibitors, rapamycin and COX-2 inhibitors having been studied in combination with OVs (Spirito et al., 2025).

Cisplatin, a platinum-based agent, induces DNA cross-linking and apoptosis and is a key drug in head and neck cancer treatment across definitive and palliative settings. Its affordability leads to widespread use in low-resource countries like India. In preclinical models, cisplatin enhances oncolytic virotherapy by reducing cytokine production, promoting viral replication, and increasing cytotoxicity. When combined with oncolytic viruses such as HSV-1, reovirus, adenovirus, and VSV, it has shown to improve therapeutic outcomes, enable dose reduction, and facilitate tumor regression. This synergy supports its potential role in combination regimens involving oncolytic viruses for more effective cancer treatment (Simpson et al., 2016).

Additionally, advances in oncolytic virotherapy have enabled the development of second-generation “armed” viruses that express therapeutic transgenes or enzymes that sensitize tumor cells to specific drugs. One such approach, gene-directed enzyme prodrug therapy (GDEPT), allows viruses to convert inactive prodrugs into toxic agents within tumor cells, enhancing chemotherapy precision while limiting systemic toxicity. These strategies can also trigger strong bystander effects, but their success depends on efficient, tumor-specific expression of the activating enzyme (Zou et al., 2020).

ICIs are being widely researched in the treatment of head and neck cancers in today’s era. CTLA-4 inhibitors (Ipilimumab) and PD-1/PD-L1 inhibitors (Nivolumab, Pembrolizumab, Atezolizumab, Durvalumab, among others) are the two main categories of ICIs out of which Nivolumab and Pembrolizumab (both PD-1 inhibitors) have been approved for the treatment of recurrent/metastatic HNSCC. ICIs are more effective against tumors with high tumor-infiltrating lymphocytes (TILs), referred to as “hot” tumors in immunology (Galon and Bruni, 2019). However, HNSCC is categorized as a “cold” tumor due to the minimal levels of TILs in the TME (Knitz et al., 2021). OVs, whether genetically modified or naturally occurring, selectively replicate within and destroy cancer cells while sparing healthy tissue. They can help overcome tumor immune resistance by attracting TILs, enhancing immune responses through tumor neoantigen release, and transforming immunologically “cold” tumors into “hot” ones (Rojas et al., 2015). The timing of ICI administration in combination therapies is critical. Some studies suggest that using anti-PD-1 alongside OVs shortly after virotherapy maintains the priming of effector T cells while preventing their exhaustion (Rojas et al., 2015).

Alongside the MASTERKEY-232 trial (T-VEC and pembrolizumab), a phase 1 trial of ONCR-177, an intratumorally administered oncolytic HSV-1, showed it to be safe and well-tolerated alone or with pembrolizumab in patients with advanced solid tumors including HNSCC. Local responses were observed at the recommended phase 2 dose, including 3 complete and 2 partial responses, all in surface lesions and in patients previously treated with ICIs. However, systemic efficacy in visceral lesions was limited (Gaspar et al., 2024). TILT-123 is an adenovirus with two potent anti-tumor cytokines (TNF-α and IL-2). A study into the use of TILT-123 and avelumab for treating melanoma and HNSCC after anti-PD-L1 therapy (AVENTIL) (NCT05222932) has been designed and is currently recruiting patients (Good Clinical Practice Network, 2025).

Radiotherapy disrupts tumor cell function and the tumor microenvironment by inducing DNA damage, particularly double-strand breaks, which are repaired via homologous recombination (HR) or non-homologous end joining (NHEJ) (O’Cathail et al., 2017). Oncolytic viruses (OVs) such as HSV-1 and adenoviruses can exploit this DNA damage response by interfering with repair proteins like the MRN complex and DNA-PKcs, enhancing viral replication. Viral proteins such as ICP0 and E4orf6 further contribute to radiosensitization by suppressing DNA repair and triggering immunogenic cell death (Hadjipanayis and DeLuca, 2005). RT also enhances OV effectiveness by increasing viral entry, gene expression, and distribution within tumors. Simultaneously, OVs stimulate anti-tumor immunity and help convert immunologically “cold” tumors into “hot” ones. When combined, RT and OVs work synergistically to amplify immune activation, improve antigen presentation, and increase tumor cell killing, offering a promising approach in cancer treatment. While several OVs, like T-VEC and CVA21, have shown promise in solid tumors, clinical studies combining OVs with radiotherapy remain limited–especially in head and neck cancers (Jayalie and Sekarutami, 2022). Such combination strategies can leverage the existing radiotherapy and chemotherapy structure while adding oncolytic viruses to the treatment armamentarium.

## Comparative advantages and limitations of OV therapy in HNSCC

Compared to tumors like melanoma and glioblastoma, where oncolytic virotherapy has achieved regulatory success and more consistent clinical outcomes, the application of OVs in HNSCC presents a unique but underdeveloped therapeutic landscape. Melanoma, characterized by high immunogenicity and pre-existing T cell infiltration, has shown more robust responses to both OVs and checkpoint inhibitors, facilitating the success of agents like T-VEC in this context (Zhang et al., 2023; Zou et al., 2020; Galon and Bruni, 2019). Glioblastoma, although less immunogenic, has benefited from recent innovations in OV design and delivery, such as Teserpaturev (Ghorab et al., 2025). In contrast, HNSCC tumors–particularly those associated with tobacco exposure–often possess an immunosuppressive microenvironment, with low TIL density and high Treg infiltration, which may blunt the immune-activating potential of OVs (Knitz et al., 2021; Cheng et al., 2021). Furthermore, hypoxic conditions in HNSCC can hinder viral replication and spread, reducing overall efficacy (Shen et al., 2006). However, HNSCC also offers certain advantages: tumors are frequently accessible for intratumoral delivery, and HPV-positive subtypes may present enhanced responsiveness to OV-induced immune priming due to pre-existing viral antigens and a more inflamed tumor microenvironment (Spirito et al., 2024). These features suggest that while biological and logistical challenges exist, HNSCC remains a rational and potentially responsive candidate for viroimmunotherapy when optimized through precision delivery and combination strategies.

## Challenges and future directions

Despite promising preclinical results and early-phase clinical outcomes, oncolytic virotherapy faces several obstacles that limit its widespread clinical application in HNSCC. One major barrier is efficient intratumoral delivery, particularly in solid tumors where the dense extracellular matrix restricts viral diffusion. This is especially relevant for HNSCC, given the region’s complex anatomical structures and lymphovascular networks. While superficial lesions can be accessed via direct injection, deep-seated or multifocal tumors require more sophisticated delivery approaches (Goradel et al., 2021).

Another challenge is tumor specificity. Although OVs exhibit preferential replication in tumor cells, off-target effects can still occur, and non-selective infection may cause toxicity in normal tissues (Kloos et al., 2015). Moreover, patients may possess pre-existing immunity against viral vectors due to natural infection or prior vaccination, leading to premature neutralization of systemically delivered OVs (Tsai et al., 2004). Several methods to circumvent this issue are under investigation, including viral cloaking with polymers, encapsulation in carrier cells, or co-administration with transient immunosuppressive agents (Cheng et al., 2021).

Tumor microenvironments are known to be hypoxic which have been found to be adversely impacting the mechanism of oncolytic viruses. Such environments, common in solid tumors like HNSCC, can hinder viral replication by suppressing oxygen-dependent cellular pathways that viruses rely on for gene expression and protein synthesis. Hypoxia also contributes to immune evasion by impairing antigen presentation and promoting the accumulation of immunosuppressive cells thus blunting the immunostimulatory effects of oncolytic viruses. Additionally, it can also modify the tumor vasculature and extracellular matrix impacting viral spread through the tumor (Shen et al., 2006). Strategies to overcome these challenges include using hypoxia-responsive viral promoters, combining OVs with agents that modulate the tumor microenvironment, or using cellular carriers to enhance delivery (Wong et al., 2010).

Efficient delivery of oncolytic viruses remains a major hurdle in treating solid tumors such as head and neck squamous cell carcinoma. The complex anatomy and extensive vascular and lymphatic networks of the head and neck region complicate local delivery, while systemic administration faces challenges of non-specificity, immune clearance, and limited tumor penetration due to the dense extracellular matrix and abnormal vasculature. Intratumoral injections may benefit superficial lesions but are impractical for deep or multifocal disease. To overcome these barriers, novel delivery strategies–such as stem cell carriers, tumor-derived microparticles, and chemokine-guided immune cells–are being explored to enhance tumor targeting and protect viral integrity (Hamada and Yura, 2020).

A critical bottleneck in OV research is the lack of large, randomized clinical trials, particularly Phase III/IV studies that evaluate long-term safety, survival benefits, and quality-of-life outcomes. Most existing trials involve small patient cohorts with heavily pre-treated or refractory disease, making generalizability difficult. Recruitment challenges, financial constraints, and regulatory complexities further delay the clinical adoption of these agents.

India carries one of the highest global burdens of HNSCC, yet lacks dedicated clinical trials investigating oncolytic virotherapy. Addressing this gap will require targeted investments, streamlined regulatory frameworks, and stronger academic-industry partnerships. Future directions should prioritize improving vector design, enhancing tumor specificity, and optimizing combination strategies. Advances in synthetic biology, personalized virotherapy, and multi-omics may support the development of tailored viral platforms. Aligning these therapies with region-specific immune profiles, viral seroepidemiology, and tumor virome characteristics could establish a foundation for precision virotherapy across cancers prevalent in low- and middle-income countries.

## Conclusion

Oncolytic virotherapy represents a novel and evolving strategy in the management of head and neck cancers, offering the unique potential to simultaneously destroy tumor cells and stimulate systemic antitumor immunity. While T-VEC remains the only US FDA-approved OV to date, numerous others–such as Oncorine, Rigvir, and Teserpaturev–are gaining regulatory traction worldwide. Clinical trials in HNSCC have demonstrated safety and local efficacy, especially when OVs are combined with immune checkpoint inhibitors, chemotherapeutic agents, or radiotherapy.

Despite these advancements, several challenges persist, including inefficient delivery, host immunity, tumor hypoxia, and limited large-scale data. Overcoming these hurdles will require innovations in viral engineering, combination treatment strategies, and patient-specific approaches. In high burden low resource settings, addressing these challenges remain critical. Continued research into virus-host dynamics, immune modulation and seroepidemiology will be key in developing successful therapies. With continued interdisciplinary research and international collaboration, oncolytic viruses may become an integral component of future multimodal regimens for head and neck cancer.
